# LanB1 Cooperates With Kon-Tiki During Embryonic Muscle Migration in *Drosophila*

**DOI:** 10.3389/fcell.2021.749723

**Published:** 2022-01-03

**Authors:** Juan José Pérez-Moreno, Carmen Santa-Cruz Mateos, María Dolores Martín-Bermudo, Beatriz Estrada

**Affiliations:** ^1^ Centro Andaluz de Biología del Desarrollo, Universidad Pablo de Olavide/CSIC/JA, Seville, Spain; ^2^ Departamento de Biología Celular, Universidad de Sevilla and Instituto de Biomedicina de Sevilla (IBiS), Hospital Universitario Virgen del Rocío/CSIC/Universidad de Sevilla, Seville, Spain; ^3^ Department of Physiology, Development and Neuroscience, University of Cambridge, Cambridge, United Kingdom

**Keywords:** myogenesis, migration, adhesion, myotendinous junction (MTJ), drosophila, laminin, Kon-tiki, NG2

## Abstract

Muscle development is a multistep process that involves cell specification, myoblast fusion, myotube migration, and attachment to the tendons. In spite of great efforts trying to understand the basis of these events, little is known about the molecular mechanisms underlying myotube migration. Knowledge of the few molecular cues that guide this migration comes mainly from studies in *Drosophila*. The migratory process of *Drosophila* embryonic muscles involves a first phase of migration, where muscle progenitors migrate relative to each other, and a second phase, where myotubes migrate searching for their future attachment sites. During this phase, myotubes form extensive filopodia at their ends oriented preferentially toward their attachment sites. This myotube migration and the subsequent muscle attachment establishment are regulated by cell adhesion receptors, such as the conserved proteoglycan Kon-tiki/Perdido. Laminins have been shown to regulate the migratory behavior of many cell populations, but their role in myotube migration remains largely unexplored. Here, we show that laminins, previously implicated in muscle attachment, are indeed required for muscle migration to tendon cells. Furthermore, we find that laminins genetically interact with *kon-tiki*/*perdido* to control both myotube migration and attachment. All together, our results uncover a new role for the interaction between laminins and Kon-tiki/Perdido during *Drosophila* myogenesis. The identification of new players and molecular interactions underlying myotube migration broadens our understanding of muscle development and disease.

## Introduction

Muscle development is a complex process where a series of cellular events need to be timely coordinated to render functional contracting muscles. First, myoblasts are specified, then they fuse with each other to form nascent myotubes, which migrate and attach to the tendons, then final differentiation takes place (Bate, 1990; Schulman et al., 2015). The formation of a stable myotendinous junction (MTJ) is key to withstand the strong forces generated by muscle contraction, and MTJ defects have been associated with myopathies in animal models and in human (Mayer et al., 1997; Bassett and Currie, 2003; Conti et al., 2008; Wang et al., 2008; Perkins et al., 2010).

*Drosophila* MTJ formation is a well-established model to study cell adhesion in organogenesis, where both the genes and cell behaviors are conserved. At late stages of muscle development, once muscle and tendons have physically contacted, final adhesion takes place by the assembly of a robust hemi-adherens junction between these cells (Schnorrer and Dickson, 2004; Schweitzer et al., 2010). This junction contains cell adhesion receptors and extracellular matrix (ECM) proteins (Valdivia et al., 2017), and the absence of these types of molecules leads to the formation of rounded muscles or myospheres. Myospheres can be observed in mutants of genes encoding cell adhesion receptors such as integrins (Martin-Bermudo et al., 1998) and Kon-tiki/Perdido (Kon) (Estrada et al., 2007; Schnorrer et al., 2007), as well as in genes encoding ECM components such as Tsp (Chanana et al., 2007; Subramanian et al., 2007), basement membrane proteins type IV collagen (Borchiellini et al., 1996) and laminins (Martin et al., 1999; Urbano et al., 2009; Wolfstetter and Holz, 2012). Although the formation of myospheres has been traditionally attributed to defects in the stabilization of the MTJ, defects in the earlier process of muscle migration towards tendon cells can also lead to the same phenotype (Kramer et al., 2001; Swan et al., 2004).

Myotube migration towards tendons depends on several factors, including the initial polarity of the muscle cell, local signals available during the migration, target recognition and terminating migration signals. It involves a cross-talk between muscles and tendons, where the latter not only serve as attachment sites but also provide guiding cues for the migrating myotube (Schweitzer et al., 2010). Then myotendinous contact and final adhesion takes place (Schnorrer and Dickson, 2004; Schweitzer et al., 2010). Although there have been shown a few genes involved in myotube migration and elongation (Maartens and Brown, 2015), very little is known about the underlying mechanisms regulating myotube migration. The conserved single-pass transmembrane proteoglycan Kon has been related not only with the assembly of the MTJ, but also with myotube migration. Muscles mutant in the gene *kon* fail to migrate in a directed manner being often lost or “perdidos” (in Spanish) and ending up as rounded unattached muscles (Estrada et al., 2007; Schnorrer et al., 2007; Pérez-Moreno et al., 2014). Kon interacts with PS2 integrin and the ECM protein Tsp in the formation of a stable muscle attachment (Pérez-Moreno et al., 2017) and it has been proposed to interact with αPS1 in the tendon (Estrada et al., 2007). Moreover, Kon contains a PDZ binding domain that forms a protein complex with the PDZ intracellular protein Grip, also involved in myotube guidance (Swan et al., 2004). However, it is still unclear which are Kon extracellular ligands during myotube migration. ECM molecules play important roles in embryonic cell migration (Walma and Yamada, 2020) but there are no ECM molecules yet identified to play a role in myotube migration.

Laminins are main basement membrane components, consisting of single α, *ß* and *γ* chains that coil to form a cross shape heterodimer, and are well-known integrin ligands (Hohenester, 2019). They are secreted and proposed to self-assemble into networks where they recruit other basement membrane components (Mouw et al., 2014). The *Drosophila* genome encodes only four laminin chains: two *α* chains (*α*1,2 and *α*3,5), one *ß* chain and one *γ* chain. These form two trimers, lamininA (*α*3,5; β1; *γ*1) and lamininW (α1,2; β1; γ1). Laminins are key regulators of embryonic morphogenesis, where they play different roles, such as migration processes. For instance, laminin *α*1,2-chain regulates embryonic tracheal migration and muscle-tendon adhesion (Martin et al., 1999), and LanA regulates axonal pathfinding (García-Alonso et al., 1996). Since laminins play a role in embryonic cell migration; are required for muscle-tendon attachment (Martin et al., 1999; Urbano et al., 2009); bind the tendon expressed *α*PS1βPS integrin (Gotwals et al., 1994), which might play a role in early events of the formation of the MTJ (Roote and Zusman, 1995; Estrada et al., 2007); and genetically interact with *Kon* (Wolfstetter and Holz, 2012), we aimed to study the potential role of laminins in myotube migration and its cooperation with Kon during this process. In particular, we analyze the role of *LanB1,* the only *Drosophila* gene encoding for the laminin *ß* subunit, present in both functional laminin *a*-*β*-*γ* heterotrimers. We show that *LanB1* plays specific roles in both muscle migration and attachment to tendon cells, as well as a strong genetic interaction between *LanB1* and *kon* during these processes. Our work suggests a key role for both muscle cell receptors and ECM components during different stages of MTJ morphogenesis.

## Material and Methods

### *Drosophila* Strains and Genetics

**FIGURE 1:**
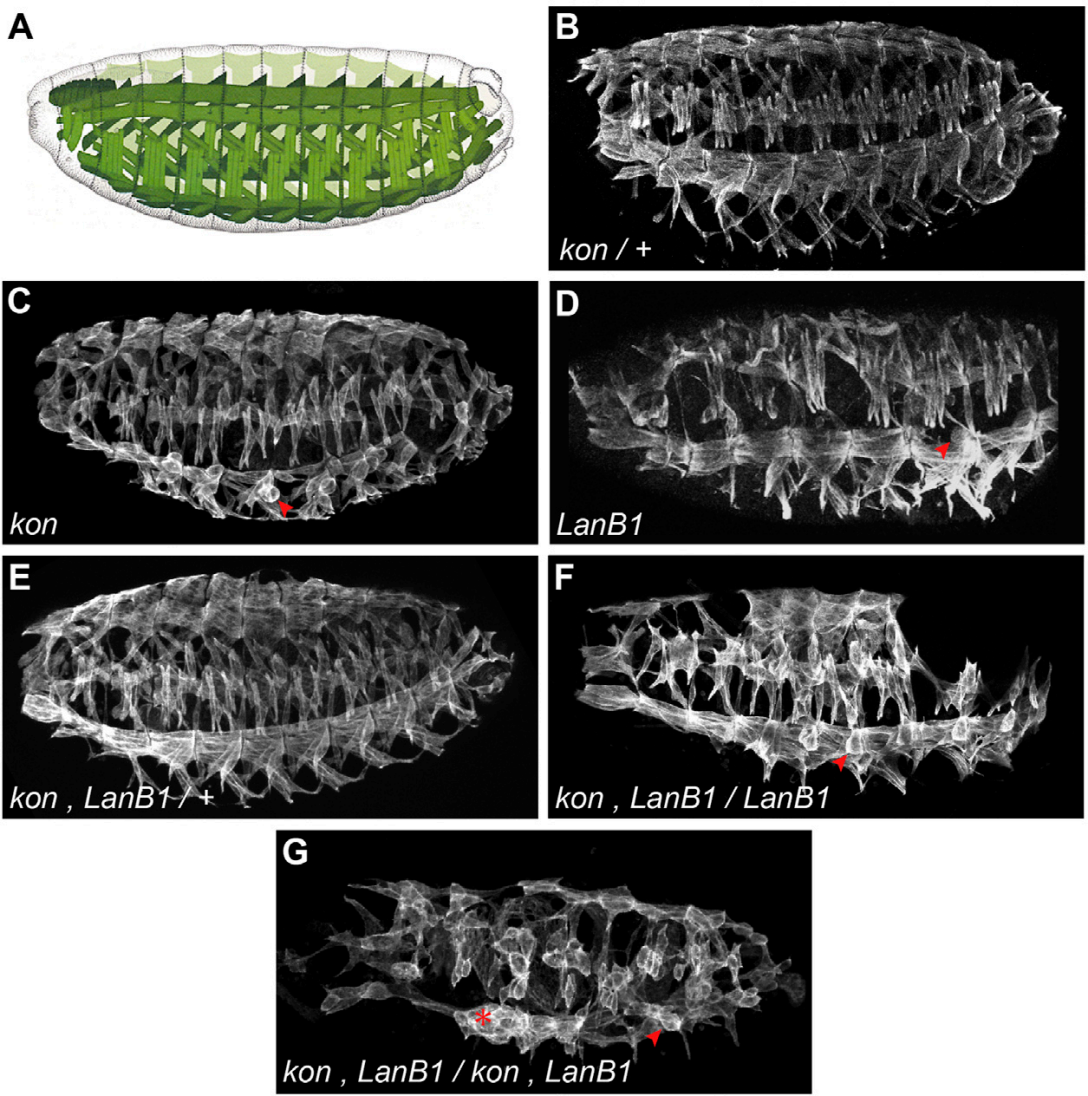
Embryonic pattern of the body wall musculature. **(A)** Scheme of the *Drosophila* muscle pattern at the end of embryogenesis (adapted from Bate and Martinez Arias, 1993). **(B–G)** Representative examples of confocal projections of stage 16 embryos. In each case, a complete penetrance of the shown phenotypes was observed [*n* = 12**(B)**; *n* = 9**(C)**; *n* = 4**(D)**; *n* = 5**(E)**; *n* = 5**(F)**; *n* = 24**(G)**]. Muscles are labeled with anti-Tropomyosin; arrowheads indicate examples of myospheres; asterisk indicates accumulation of detached muscles.

The following stocks were used: *kon* (*kon* ^*F1-3*^) (Estrada et al., 2007); *LanB1* ^*DEF*^ and *LanB1* ^*1P3*^ (Urbano et al., 2009); *5053-GAL4* (Bloomington *Drosophila* Stock Center: 2702) (Swan et al., 2004); *UAS-src:GFP* (Lesch et al., 2010). The *CyO, twist-GAL4, UAS-2EGFP* (Halfon et al., 2002), *CyO, ftz-LacZ* (Knoblich and Lehner, 1993), and *CyO*, *act:GFP* (Bloomington *Drosophila* Stock Center) balancer chromosomes, were used to identify homozygous mutants. In both Figure 1 and Figure 3, we used the *kon* ^*F1-3*^ *, LanB1* ^*DEF*^ (*Rec8*) recombinant chromosome to analyze double *kon* and *LanB1* mutation.

### Embryo Immunohistochemistry and Microscopy

Embryo antibody stainings were carried out as described previously (Carmena et al., 1998). The following primary antibodies were used: 1:5,000 rabbit anti-*β*-galactosidase (Cappel, 55,976); 1:5,000 rabbit anti-GFP (Invitrogen, A-6455), 1:5,000 mouse anti-GFP (Invitrogen, A-11120), and 1:400 rat anti-Tropomyosin (Babraham Bioscience Technologies, MAC141). The following secondary antibodies were used: 1:200 goat anti-rabbit-Cy2 (Jackson, 111-225-144), 1:200 goat anti-rat-Cy5 (Jackson, 112-175-143), 1:200 goat anti-mouse-488 (Life Technologies, A-1101), and 1:200 goat anti-rabbit-Cy5 (Jackson, 111-175-144). Confocal images were obtained using SP2 and Stellaris five confocal microscopes from Leica, and processed with Adobe Illustrator and ImageJ.

### Data Analysis

The identification of specific embryonic stages was performed by following previous characterization (Campos-Ortega and Hartenstein, 1997). Stage 13–14 embryos showed head involution with retracting clypeolabrum, germ band retraction, yolk sac protruding dorsally and dorsal closure of midgut and epidermis; Stage 15 embryos showed retracted clypeolabrum, completion of dorsal closure, and growth of the hindgut; Stage 16 embryos showed midgut constrictions and final pattern of somatic musculature was developed. In *LanB1* mutants there is a failure in midgut constriction so that staging of these embryos was based on the other morphologies. Quantification of phenotypes in VL1 muscle morphology was performed by counting the number of each type of phenotype per embryo. Image analysis was performed from both confocal z-stacks and maximal projections using ImageJ software.

Our VL1 muscles data consisted of relative proportions of *WT* and several types of muscle phenotypes across genotypes. Since the relative proportion of muscles in each category was not statistically independent from each other, we first conducted an overall compositional data analysis (Aitchison, 1982) using the package *CoDaPack* (Comas and Thió-Henestrosa, 2011). Visual inspection of the data distribution across the tetrahedron (Figure 3A) suggested that genotypes differed markedly in the proportion of abnormal muscles, which was confirmed statistically by a Multiple Analysis of Variance (MANOVA). Confirmed these overall differences across genotypes, we then tested for statistical differences between genotypes or within genotypes across stages in the proportion of specific muscle phenotypes. We tested such comparisons fitting Generalized Linear Models with a binomial error distribution and a logit link function, where the dependent variable was *number of muscles with a given phenotype vs total number of muscles* scored in a given embryo.

## Results

### *LanB1* and *Kon* Interact Genetically During the Development of the Myotendinous Junction in *Drosophila* Embryos

To study the role of laminins and their interaction with *kon*, during the development of the MTJ, we first studied the pattern of the body wall musculature at the end of embryogenesis, stage 16 (st. 16) (Figure 1A), in both control and mutant embryos. To study *kon* function, we analyzed embryos for the null mutant allele *kon* ^*F1-3*^ (hereafter referred as *kon*). In agreement with previous findings (Estrada et al., 2007; Schnorrer et al., 2007), we found that, while control embryos (*kon*/+) did not show any defect in the musculature (Figure 1B), some muscles from *kon* embryos, particularly the ventral-longitudinal ones (VL), formed myospheres, a term used to describe rounded shape muscles due to detachment from their tendon cells (Wright, 1960) (arrowhead in Figure 1C). To study laminin function, we analyzed embryos carrying a deficiency that eliminates the only *Drosophila* gene encoding the laminin *ß*-subunit, *LanB1*, hereafter referred as *LanB1* embryos (Figure 1D). Similar to *kon* embryos, *LanB1* mutant muscles also formed myospheres, particularly the VL ones (Urbano et al., 2009) (arrowhead in Figure 1D). Since loss of function of either *kon* and *LanB1* showed myospheres, we studied their cooperation during MTJ development by performing genetic interaction experiments. To do this, we generated flies with a recombinant chromosome for both mutations. Double *kon, LanB1* heterozygous mutants showed a wild-type muscle pattern (Figure 1E), suggesting that a single functional allele for each gene is enough to properly establish the muscle pattern. However, we found that the muscle detachment phenotype of *LanB1* embryos that were also *kon* heterozygotes, was dramatically enhanced compared with either *kon* or *LanB1* single mutants (Figure 1F). Moreover, complete loss of both *kon* and *LanB1* (hereafter referred as *kon, LanB1* embryos) led to a generalized presence of myospheres (Figure 1G) (quantification below). The high number of detached muscles in the mutant combination *kon, LanB1* (either partial or complete), resulted in the formation of gaps in the muscle pattern (Figures 1F,G and Supplemetary Figure S1). Since *LanB1* deficiency also removes the 5’ UTR of the adjacent *CG72143* gene, we validated our results by recombining *kon* ^*F1-3*^ with a null allele for *LanB1, LanB1* ^*1P3*^ (Urbano et al., 2009). Consistently, these recombinant mutant embryos showed the same phenotype as the *kon, LanB1* embryos (Supplemetary Figure S1). Altogether, this data supports a cooperative role for *LanB1* and *kon* in the formation of the MTJ.

### *LanB1* is Required for Muscle Migration Towards Tendon Cells

**FIGURE 2:**
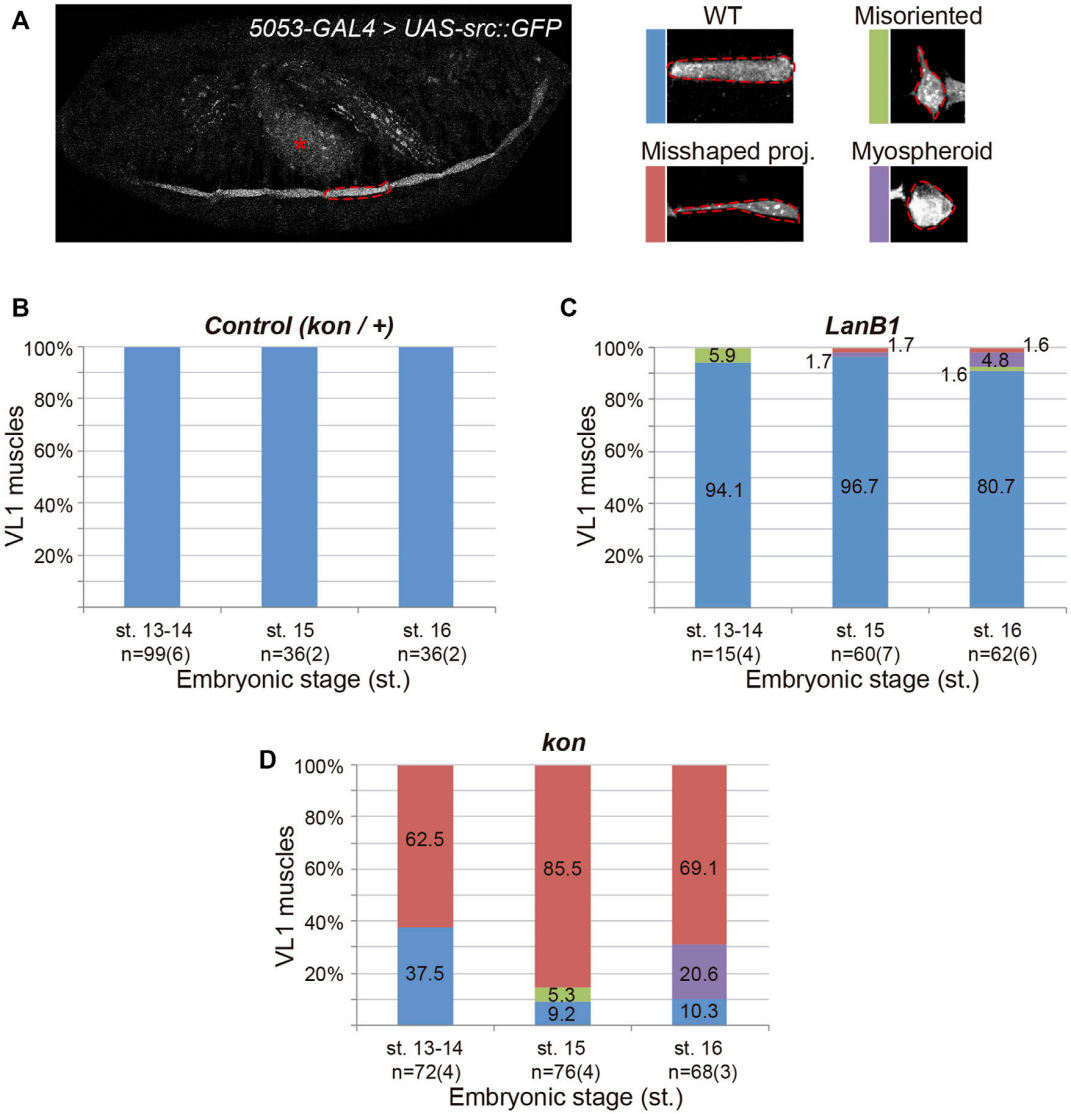
Development of ventral-longitudinal muscle 1 (VL1). **(A)** Left panel shows a representative example of the confocal projection in a stage 16 embryo. *5053-GAL4* expresses the plasma membrane marker src:GFP in the VL1 muscle of each segment (dotted line indicates one example) and the gut (asterisk). Right panels show representative examples of the different morphological phenotypes observed in VL1 muscle for the genotypes indicated. **(B–D)** Quantification of the proportion of VL1 muscles according to their morphology at different embryonic stages for the indicated genotypes. Bar colors indicate VL1 muscle phenotypes: WT, blue; Misoriented, green; Misshaped projection, red; Myospheroid, purple. Sample size indicates the total number of muscles over the number of embryos indicated between parentheses.

The muscle detachment phenotype observed at the end of embryogenesis in *LanB1* embryos (Figure 1D) (Urbano et al., 2009) might be caused by defects in either muscle guidance and/or muscle attachment to tendon cells. To distinguish between these two possibilities, and to further characterize the role of *LanB1* in the formation of the MTJ, we characterized muscle migration and attachment in both control and *LanB1* embryos. Previous studies have shown that as muscles migrate towards their tendon cells they extend projections towards future attachment positions (Volk, 1999; Schnorrer and Dickson, 2004). The visualization of the complete body wall musculature (Figure 1) complicates the analysis of the morphology of individual muscles as they migrate, as well as their cellular protrusions. To solve this, we decided to label just one specific muscle, VL1, using *5053-GAL4* driver (Swan et al., 2004) to express a GFP-tagged plasma membrane marker, *src:GFP* (Lesch et al., 2010) (Figure 2). *wild type (wt)* muscles (Figures 2A,B) (Schnorrer et al., 2007) first project protrusions anteriorly towards their future attachment positions at the anterior segment border (st. 13–14). The posterior end does not migrate, as it is already placed near the posterior segment border, the other attachment point. Then muscles initiate (st. 15) and stabilize (st. 16) the attachment to tendon cells (Figure 2A). In *LanB1* embryos (Figure 2C), at early stages (13–14), 6% of the VL1 muscles showed misoriented projections, that is, muscles with projections oriented perpendicularly to the normal posterior to anterior direction of migration (Figure 2A). At st. 15, *LanB1* loss caused a few cases of myospheroid-shape muscles (rounded, and detached muscles) (2%) and of muscles with misshaped projections (2%), muscles whose projections were properly oriented but longer than normal, and presenting a small contact surface with the attachment site (Figure 2A), which reflects a defect in the initial contact between muscle and tendon cells. Moreover, at st. 16, 5% of the muscles were myospheres, and some muscles showed misoriented or misshaped projections (2% each). Our data suggests that *LanB1* regulates muscle targeting to tendon cells at early stages and stabilization of muscle attachments later in embryogenesis. The fact that *LanB1* muscles send projections suggests that LanB1 is not required for the overall formation of filopodia during muscle migration, although we cannot distinguish if they are intact at this resolution.

As in *LanB1* embryos, we had observed that *kon* embryos also showed myospheres at the end of muscle development (Figure 1) (Estrada et al., 2007; Schnorrer et al., 2007). However, the specific analysis of VL1 muscle morphology at early and late stages revealed striking differences between both genotypes (Figures 2C,D) (Estrada et al., 2007; Schnorrer et al., 2007). At st. 13-14, *kon* mutants showed a remarkable proportion of muscles with misshaped projections (63%), already indicating an altered ability to initiate contacts with tendon cells. However, unlike *LanB1* mutants, loss of *kon* did not produce muscles with misoriented projections at these early stages (Figure 2D) (Estrada et al., 2007; Schnorrer et al., 2007). Later, at st. 15, *kon* embryos showed an increase in the number of misshaped projections (from 63 to 86%), supporting the known role of *kon* in mediating muscle-tendon attachment. Remarkably, *kon* mutants also showed some misoriented muscles at this stage (5%), likely consequence of failed attachment, as previously reported (Schnorrer et al., 2007). Finally, at st. 16, 69% of *kon* mutants showed misshaped projections and 21% myospheres, indicating respectively incomplete and failed muscle attachments. Together, this data supports that the myospheres observed in late *kon* embryos could be due to defects in muscle-tendon migration and/or attachment formation, while LanB1 additionally showed a specific role in projection orientation at the early stages of muscle migration.

### *Kon* and *LanB1* Cooperate in Muscle Guidance and Attachment to Tendon Cells

**FIGURE 3:**
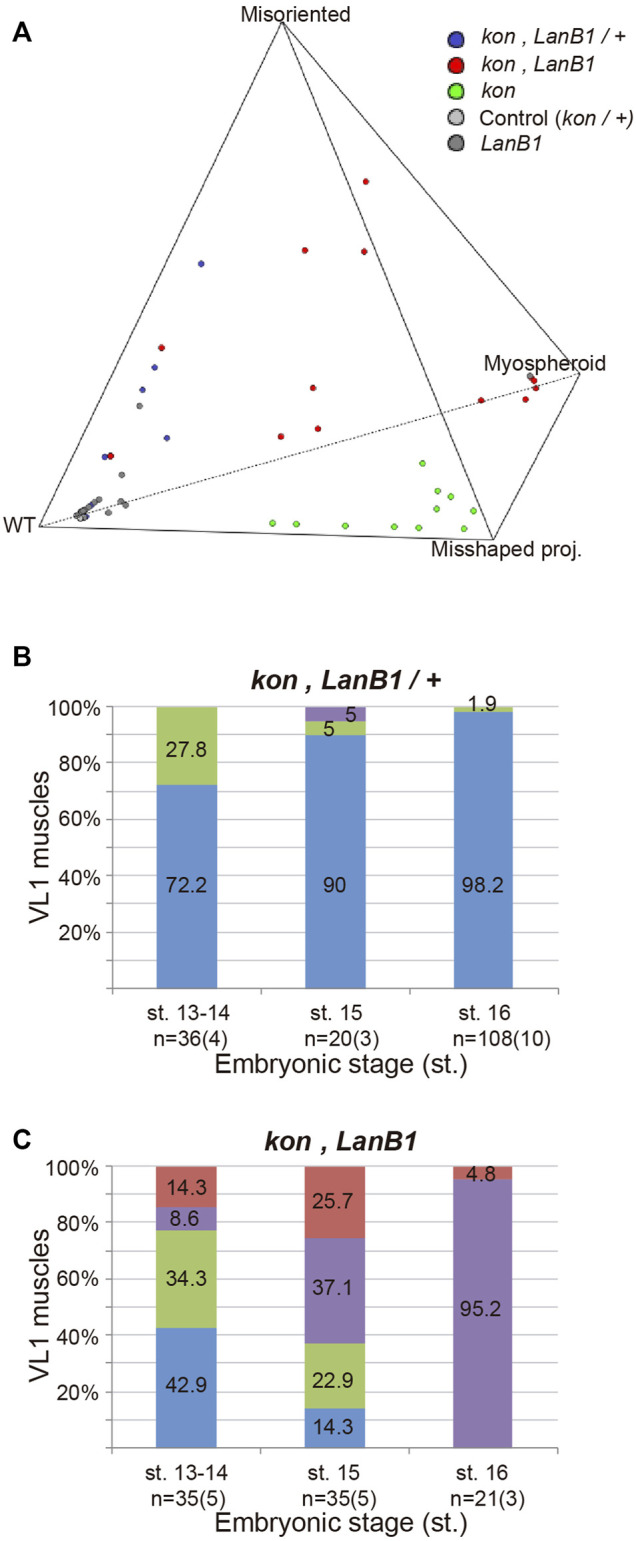
*LanB1* and *kon* interaction during the development of the VL1 muscle. **(A)** Quaternary plot showing the embryo distribution among genotypes according to the morphology of their muscles. Multivariate analysis of variance (MANOVA) statistical test, *p* < 0.00001. **(B,C)** Quantification of the proportion of VL1 muscles according to their morphology at different embryonic stages for the indicated genotypes. Bar colors indicate VL1 muscle phenotypes: WT, blue; Misoriented, green; Misshaped projection, red; Myospheroid, purple. Representative examples of these phenotypes are shown in Figure 2. Sample size indicates the total number of muscles over the number of embryos indicated between parentheses. Binomial distribution test was performed for pairwise comparisons: misoriented muscles at st. 13, 14 between *LanB1* and *kon, LanB1/+*. *p* = 0.046; misoriented muscles at st. 13, 14 between *kon, LanB1* and *kon, LanB1/+*. *p* = 0.55; total abnormal muscles at st. 13, 14 between *kon, LanB1* and *kon*. *p* = 0.60; total abnormal muscles at st. 15 between *kon, LanB1* and *kon*. *p* = 0.43.

To better understand the specific role of *LanB1* and *kon* and their interaction, we first analyzed, independently of the stage, VL1 muscle morphology in different dosages of mutant alleles for *LanB1* and *kon*. The spatial distribution of the observed muscles with different phenotypes within a polyhedron showed that there is a statistically significant difference in their distribution among genotypes. Specifically, loss of one or two copies of *LanB1* in a *kon* background caused the displacement of the *kon* phenotypes towards the misoriented muscle morphology, suggesting an interaction between *LanB1* and *kon* in muscle migration and attachment formation. This statistical analysis (Figure 3A and Supplementary Movie) (see Material and Methods for more details) allowed us to carry out the specific pairwise comparisons of the phenotypes among genotypes in different stages.

At st. 13-14, we observed that while embryos with only one functional copy of *kon* showed no alterations in muscle development (Figure 2B), the additional loss of one *LanB1* allele (double heterozygous) (Figure 3B) showed around one third (28%) of all muscles with misoriented projections. Interestingly, this was significantly higher than what we found in either *LanB1* (6%) or *kon* (0%) single mutant embryos (Figures 2C,D), supporting a cooperation between both *kon* and *LanB1* in muscle targeting. Moreover, *kon, LanB1* mutants (Figure 3C), presented a similar amount of misoriented projections than double heterozygote mutants (Figure 3B; 34 and 28% respectively), but the former additionally showed misshaped muscles and myospheres (likely unable to send projections). Together, this resulted in that more than the half (57%) of the muscles in *kon*, *LanB1* embryos presented some type of muscle phenotype at early stages. Therefore, our data supports a role for *LanB1* in muscle guidance and its cooperation with *kon* in this process.

At st. 15, comparing *kon*, *LanB1* (Figure 3C) with *kon* (Figure 2D) embryos, they both showed a similar proportion of total number of muscles with any of the described phenotypes (86 and 91% respectively), while this proportion was only 3% in *LanB1* mutants (Figure 2C). However, *kon*, *LanB1* mutants showed 37% myospheroids and 23% misoriented muscles, phenotypes which were respectively non-observed or milder (5%) in *kon* mutants. Therefore, the early formation of myospheres and the relatively high number of misoriented muscles in *kon*, *LanB1* embryos, suggest a cooperation between both genes to regulate the later stages of muscle migration and the attachment formation between muscle and tendon cells.

At st. 16, all the muscles were affected in *kon*, *LanB1* mutants (Figure 3C), where 95% were myospheres and 5% misshaped projections. The remarkable difference of the myospheres/misshaped projections ratio between *kon* (21/70) (Figure 2D) and *kon, LanB1* mutants (95/5) (Figure 3C), supports a strong cooperation between *kon* and *LanB1* in the stabilization of the myotendinous junction.

### Kon is Not Essential for LanB1 Localization at the MTJ

**FIGURE 4:**
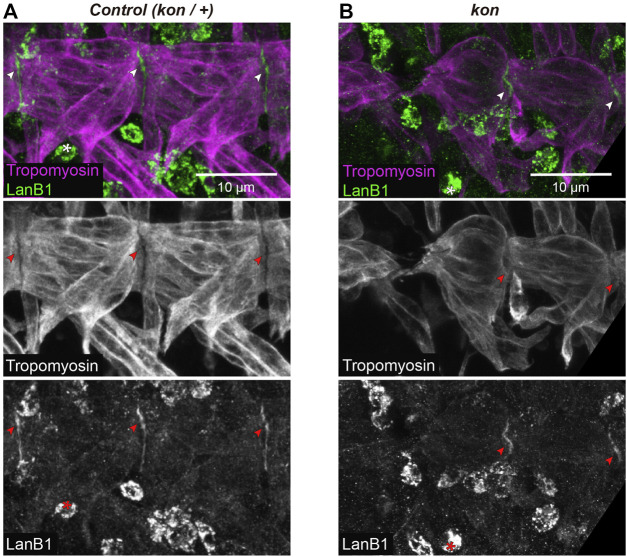
LanB1 distribution at the MTJ. **(A,B)** Representative examples of confocal projections of st. 16 embryos, where the VL muscles of two hemisegments are shown. Muscles are labeled with anti-Tropomyosin; arrowheads indicate MTJ regions (intersegmental regions) showing LanB1 enrichment; asterisks indicate haemocytes. See Supplementary Figure S2 for a detailed view of the corresponding confocal sections.

The transmembrane proteoglycan Kon is a cell adhesion receptor that mediates the interaction between the muscle cell and the ECM (Estrada et al., 2007; Schnorrer et al., 2007; Pérez-Moreno et al., 2017). Therefore, the observed cooperation between *kon* and *LanB1* during the formation of the MTJ might suggests a potential role for Kon in localizing LanB1 at the MTJ. As previously reported in *wt* condition (Urbano et al., 2009; Wolfstetter and Holz, 2012), we observed that control embryos showed LanB1 enrichment at the MTJ (arrowheads in Figure 4A and Supplementary Figure S2A). In *kon* embryos, despite the decreased MTJ surface of attachment of VL muscles, we still detected accumulation of LanB1 where muscles were attached (arrowheads in Figure 4B and Supplementary Figure S2B). This result indicates that Kon is not essential to recruit LanB1 at the MTJ.

## Discussion

The development of the MTJ requires cell adhesion receptors and ECM components (Maartens and Brown, 2015; Valdivia et al., 2017). This is supported by studies, mainly in *Drosophila*, showing that the loss of these types of molecules leads to MTJ disruption. The physical interaction between cell adhesion receptors, mostly integrins, and the ECM is well-known, as well as the requirement of this interaction in multiple cellular and developmental events (Walma and Yamada, 2020). However, the specific contribution of each of these components and interactions between them to the different cellular processes underlying MTJ development, such as migration, recognition and attachment, remains largely unknown. Here, we show that laminins, previously involved in muscle attachment, are also required for proper muscle migration to the tendon cells. Furthermore, our results support that *kon* interacts with laminins both during migration and attachment.

It has been reported that *kon* and *LanB2* interact genetically during the MTJ formation (Wolfstetter and Holz, 2012). We extended this analysis by exploring the interaction between *kon* and *LanB1*. Remarkably, we observed that double loss of *kon* and *LanB1* caused a more dramatic disruption of the body wall musculature than the previously reported in *kon, LanB2* mutants. The secretion of an individual laminin subunit has been only observed *in vitro* (Yurchenco et al., 1997), being widely studied that laminins are only secreted and functional as heterotrimers *in vivo* (Hohenester, 2019). Therefore, the different grade of disruption of the muscle pattern observed between *kon, LanB2* mutants (Wolfstetter and Holz, 2012) and *kon, LanB1* mutants (this work) might be due to differences in the genetic background of both studies. Here, we validated our data by analyzing the genetic interaction between *kon* and two different mutants for *LanB1* (*LanB1* ^*DEF*^ and *LanB1* ^*1P3*^).

Beyond its general role in MTJ formation, it was unknown whether laminin (and other ECM components) only accumulates at the MTJ to form and/or stabilize the junction, or whether it also participates during the earlier process of muscle guidance. To study this, here, we explored the effect of *LanB1* loss during MTJ development. First, we observed a requirement of LanB1 at the early stages of muscle development, supported by the presence of some misoriented muscles at stage 13–14 in *LanB1* mutants. The inability to polarize filopodia in the right direction could be due to a failure in sensing guidance cues, and it also suggests that LanB1 is not required for filopodia formation. Similarly, our previous studies have shown that while haemocytes from *LanB1* embryos can form protrusions they are not orientated in the direction of migration. Formation and stabilization of lamellipodia play a critical role in achieving directionally persistent migration in cell culture (Petrie et al., 2009). As we showed that haemocytes produced their own laminins, this led us to propose a role for laminins in reinforcing directional migration by stabilizing cellular protrusions locally. In the future, it will be interesting to analyse whether muscles can also produce their own laminins to enhance directional migration.

In contrast to the muscle guidance defects observed in *LanB1* mutants, early *kon* embryos (stage 13–14) only showed misshaped projections, what suggests that Kon plays a role in muscle-tendon recognition/attachment (Estrada et al., 2007; Schnorrer et al., 2007). Although *kon* embryos showed misoriented projections at stage 15, this might be a consequence of the failed muscle-tendon recognition/attachment observed at earlier stages. The genetic interaction observed between *kon* and *LanB1* further supports a role for both LanB1 and Kon in muscle guidance. It remains open, however, whether LanB1 only plays a role in muscle guidance or whether it has an additional role in the stabilization of the attachment. The observation of myospheres at late stages in *LanB1* embryos could be partially due to a LanB1 role during muscle guidance, but also to an additional role in muscle-tendon adhesion. However, the fact that practically all muscles form myospheres in late *kon*, *LanB1* embryos, cannot be only correlated with the proportion of misoriented muscles observed earlier in the same embryos. Since there are no muscle contractions at st. 15 and only isolated brief muscles twitches by the end of st.16 (Perenau et al., 2007; Crisp et al., 2008), the observed myospheres in our study are most likely due to failed formation of the MTJ and not to a contraction-derived detachment. Therefore, our data suggests that misoriented muscles, muscles with misshaped projections and also some muscles with no phenotype, detached from tendon cells in late *kon*, *LanB1* embryos, supporting a role for LanB1 in stabilizing the MTJ. This potential role is also supported by the progressive accumulation of LanB1 in the mature MTJ from st. 14 to 16 (Martin et al., 1999), and by the fact that LanA is required for the adhesion of the basement membrane to the muscle surface in the formation of a similar hemi-adherens junction, the one in the neuromuscular junction (Prokop et al., 1998; Tsai et al., 2012).

Despite the requirement of both LanB1 and Kon during muscle development, we propose they are likely acting in parallel. The strong genetic interaction observed suggests they are neither upstream/downstream of each other in the same pathway nor working together as ligand and receptor. In addition, we observed that LanB1 localization is not regulated by Kon. However, they still might be part of the same molecular complex, where loss of different components could affect its functionality. In fact, it has been shown that the Kon orthologue, NG2, can physically interact *in vitro* with laminin (Burg et al., 1996).

Cell receptors can influence the ECM which in turn feedback on cell adhesion through the receptors (Maartens and Brown, 2015). In fact, later in MTJ development, Kon localizes the ECM protein Tsp and enhances PS2 integrin adhesion (Pérez-Moreno et al., 2017). Both LanB1 and Kon have been shown to interact with integrins (Estrada et al., 2007; Maartens and Brown, 2015) but how these protein complexes are coordinated in regulating the intricate molecular mechanisms underlying muscle guidance and adhesion to the tendons needs to be further elucidated.

## Data Availability

The original contributions presented in the study are included in the https://doi.org/10.17605/OSF.IO/JWHZU, further inquiries can be directed to the corresponding author.
