# Corrigendum: LanB1 Cooperates With Kon-Tiki During Embryonic Muscle Migration in *Drosophila*

**DOI:** 10.3389/fcell.2022.887432

**Published:** 2022-04-13

**Authors:** Juan José Pérez-Moreno, Carmen Santa-Cruz Mateos, María Dolores Martín-Bermudo, Beatriz Estrada

**Affiliations:** ^1^ Centro Andaluz de Biología del Desarrollo, Universidad Pablo de Olavide/CSIC/JA, Seville, Spain; ^2^ Departamento de Biología Celular, Universidad de Sevilla and Instituto de Biomedicina de Sevilla (IBiS), Hospital Universitario Virgen del Rocío/CSIC/ Universidad de Sevilla, Seville, Spain; ^3^ Department of Physiology, Development and Neuroscience, University of Cambridge, Cambridge, United Kingdom

**Keywords:** myogenesis, migration, adhesion, myotendinous junction, drosophila, laminin, kon-tiki, NG2

The original Funding and Acknowledgment sections were not formatted according to the institution’s guidelines. This has now been rectified and the paragraph below replaces the previous version:

**ACKNOWLEDGMENTS**

This publication was funded by Grant IJC2019-038819-I, funded by MCIN/AEI /10.13039/501100011033. JP-M acknowledges the support from Grant IJC2019-038819-I; MCIN/AEI /10.13039/501100011033 and Proyecto de Excelencia of the Consejería de Economía, Innovación, Ciencia y Empleo, Junta de Andalucía (PO9-CVI-5058); CS-C acknowledges the support from CSIC (JAE intro 08-01103); MM-B acknowledges the support from Proyecto de Excelencia, Junta de Andalucía (PO9-CVI-5058) and Proyecto del Ministerio de Ciencia e Innovación (PID2019-109013GB-100); BE acknowledges the support from the Ramón y Cajal program, Proyecto del Ministerio de Economía y Competitividad (BFU2008-036550, BFU2011-26745), University of Pablo de Olavide and University of Seville; We thank the Bloomington Stock Center for fly stocks and Ivan Gomez-Mestre for help with the statistical analysis.

The authors apologize for this error and state that this does not change the scientific conclusions of the article in any way. The original article has been updated.

