# Supplementary figures and images for: LanB1 Cooperates With Kon-Tiki During Embryonic Muscle Migration in Drosophila
Source: Front Cell Dev Biol. 2022 Jan 3;9:749723. doi: 10.3389/fcell.2021.749723 (PMC8762229; doi:10.3389/fcell.2021.749723)

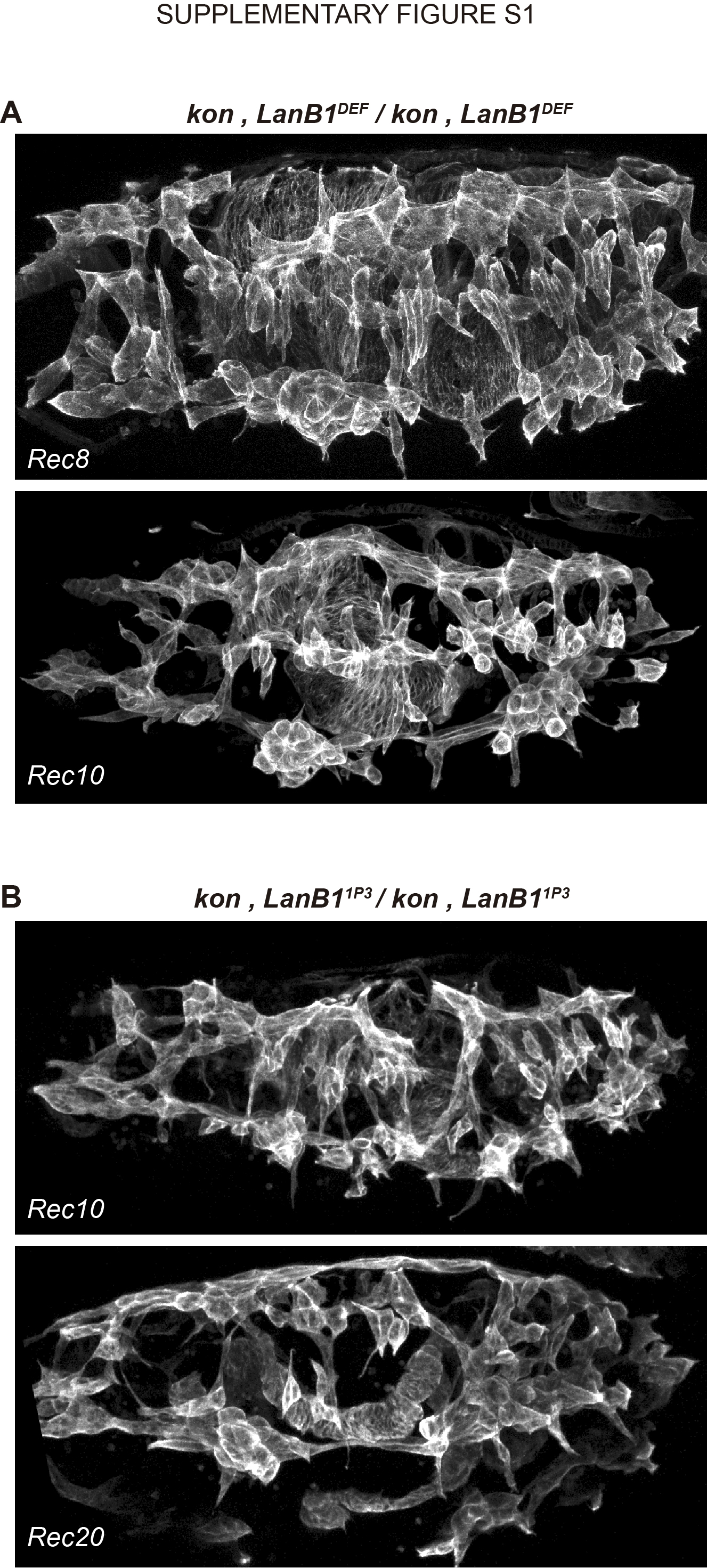

Supplement: Supplementary file 2 [file Image1.JPEG]

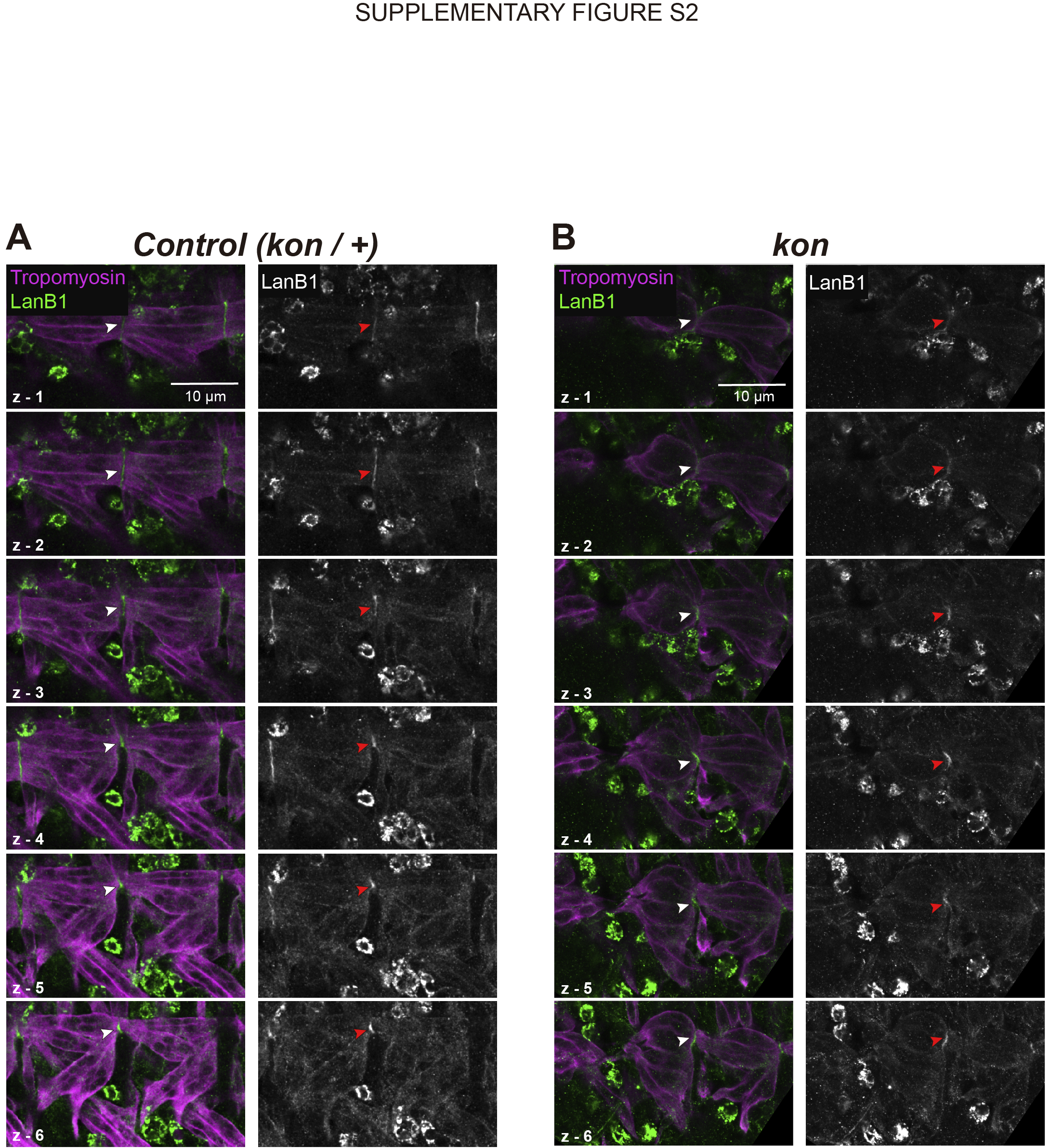

Supplement: Supplementary file 3 [file Image2.JPEG]
